# Ultra-high-resolution 3D imaging of atherosclerosis in mice with synchrotron differential phase contrast: a proof of concept study

**DOI:** 10.1038/srep11980

**Published:** 2015-07-13

**Authors:** Gabriele Bonanno, Simone Coppo, Peter Modregger, Maxime Pellegrin, Annina Stuber, Marco Stampanoni, Lucia Mazzolai, Matthias Stuber, Ruud B. van Heeswijk

**Affiliations:** 1 Department of Radiology, University Hospital (CHUV) and University (UNIL) of Lausanne, Switzerland; 2 Center for Biomedical Imaging (CIBM), Lausanne, Switzerland; 3 Swiss Light Source, Paul Scherrer Institut, Villigen, Switzerland; 4 Division of Angiology, University Hospital of Lausanne (CHUV), Switzerland; 5 Institute for Biomedical Engineering, University and ETH Zürich, Zürich

## Abstract

The goal of this study was to investigate the performance of 3D synchrotron differential phase contrast (DPC) imaging for the visualization of both macroscopic and microscopic aspects of atherosclerosis in the mouse vasculature *ex vivo*. The hearts and aortas of 2 atherosclerotic and 2 wild-type control mice were scanned with DPC imaging with an isotropic resolution of 15 μm. The coronary artery vessel walls were segmented in the DPC datasets to assess their thickness, and histological staining was performed at the level of atherosclerotic plaques. The DPC imaging allowed for the visualization of complex structures such as the coronary arteries and their branches, the thin fibrous cap of atherosclerotic plaques as well as the chordae tendineae. The coronary vessel wall thickness ranged from 37.4 ± 5.6 μm in proximal coronary arteries to 13.6 ± 3.3 μm in distal branches. No consistent differences in coronary vessel wall thickness were detected between the wild-type and atherosclerotic hearts in this proof-of-concept study, although the standard deviation in the atherosclerotic mice was higher in most segments, consistent with the observation of occasional focal vessel wall thickening. Overall, DPC imaging of the cardiovascular system of the mice allowed for a simultaneous detailed 3D morphological assessment of both large structures and microscopic details.

Atherosclerosis is a chronic and systemic disease, and over time several animal models have been developed to study its mechanisms and progression^1^,^2^. The most common experimental model of atherosclerosis is the apolipoprotein-E-knockout (ApoE^−/−^) mouse^3^. Microscopic details of the atherosclerotic plaques in these mice are commonly investigated *post-mortem* with histology^4^, while macroscopic aspects have been studied *in vivo* and longitudinally with biomedical imaging techniques such as magnetic resonance imaging (MRI)^5^ and computed tomography (CT)^6^,^7^.

Biomedical microscopic CT (micro-CT)^8^ has been developed to “fill the gap” between the macroscopic biomedical imaging techniques and histology of mice, and can be used to investigate three-dimensional (3D) structures *post mortem* at resolutions that approach those of histology. The contrast in micro-CT is based on the attenuation of X-rays in tissues, which means that it can for example be used to detect calcifications^9^, but it is relatively poor between soft tissues. Alternatively, CT contrast can be based on the X-ray refractive index of the tissue. This results in phase contrast^10^, which is much higher between soft tissues than attenuation contrast. However, commonly used micro-CT scanners do not generate sufficiently parallel (collimated) and homogeneous (monochromatic) X-rays to produce biomedically useful phase contrast images at microscopic spatial resolutions. A biomedical imaging technique that combines phase contrast with micrometer resolutions may therefore open up new avenues for the investigation of atherosclerosis in mouse models.

To address such needs, several imaging techniques have been developed that make use of synchrotron radiation^11^. A synchrotron essentially is a cyclical particle accelerator that consists of linear stretches linked by bending magnets, at which highly collimated X-ray beams are generated. These X-ray sources can be used to produce 3D images at a resolution down to 1 μm^12^, and synchrotron phase-contrast CT has for example been applied to characterize human atherosclerotic plaque *ex vivo*^13^. A more recently developed synchrotron imaging technique is differential phase contrast (DPC) imaging^14^, which makes use of two stepped gratings to perform interferometry with a very high soft-tissue contrast^15^. Such DPC imaging has for example been applied to detect amyloid plaques in the mouse brain^16^ and to improve tumor detection in human breast tissue^17^.

The goal of this study was therefore to advance mouse atherosclerosis and cardiovascular imaging by investigating the use of DPC imaging for the visualization of the 3D structure of both microscopic and macroscopic aspects of atherosclerosis in mice for the first time.

## Results

### Differential phase contrast of the whole mouse heart

**Figure 1:**
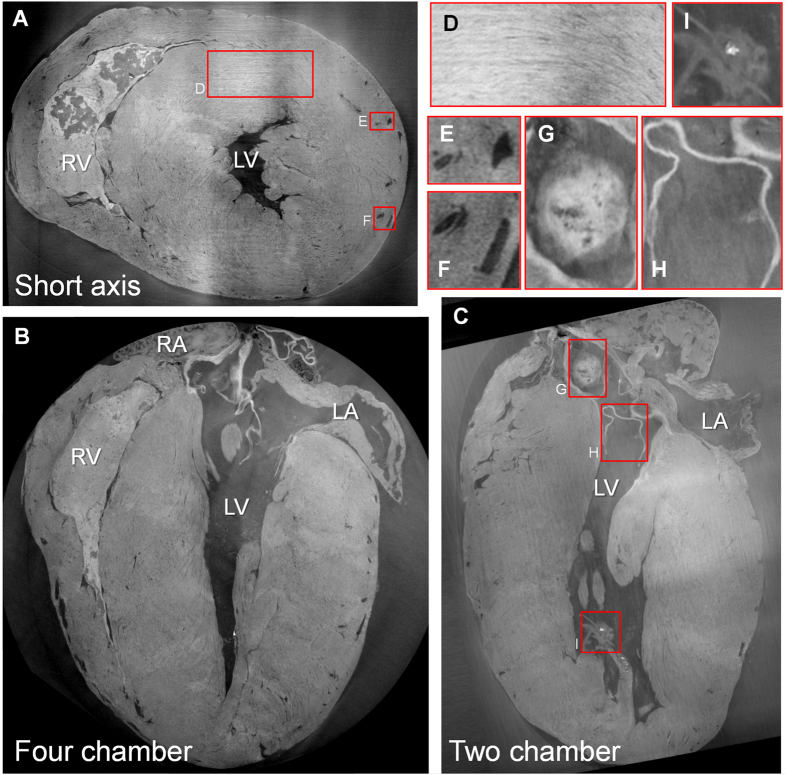
Three-dimensional DPC imaging of an *ex-vivo* ApoE^−/−^ mouse heart. (**A**–**C**) The three principal axes of the heart give a clear overview of the global structure of the heart. The enlarged insets demonstrate the excellent contrast for the visualization of smaller structures such as the orientation of the myocytes (**D**), arteries, their walls and veins (**E**,**F**), part of an atherosclerotic plaque in the aortic sinus (**G**), valves (**H**) and even small structures in the papillary muscles (**I**). Since the left system was flushed with saline after sacrifice, a blood clot was present in all right ventricles (RV). However, no blood clots were observed in the coronary arteries and veins (E,F), suggesting that the saline flush passed through the entire myocardial vascular network. Minor non-physiological low-frequency fluctuations in image intensity were visible on a macroscopic scale (A–C), but these did not impact the contrast and visibility of the macroscopic or microscopic structures. RV = right ventricle, LV = left ventricle, RA = right atrium, LA = left atrium

The DPC imaging protocol resulted in high-resolution and high-contrast overviews of the mouse hearts. The entire heart including the ventricles and atria could be inspected, as well as small structures such as the coronary artery vessel walls, atherosclerotic plaques in the aortic sinus and all valves (Fig. 1).

The orientation of the myocytes could be observed throughout the heart (Fig. 1D), while the valves (Fig. 1H) including the chordae tendineae down to their attachment to the papillary muscles had high contrast and were easily visible, despite diameters as low as 20–30 μm.

Several small (diameter 20–65 μm) bright structures were observed in the lower papillary muscles of the ApoE^−/−^ mice, which may be attributed to microcalcifications (Fig. 1I).

### Comparison of DPC images to histological ground truth

**Figure 2:**
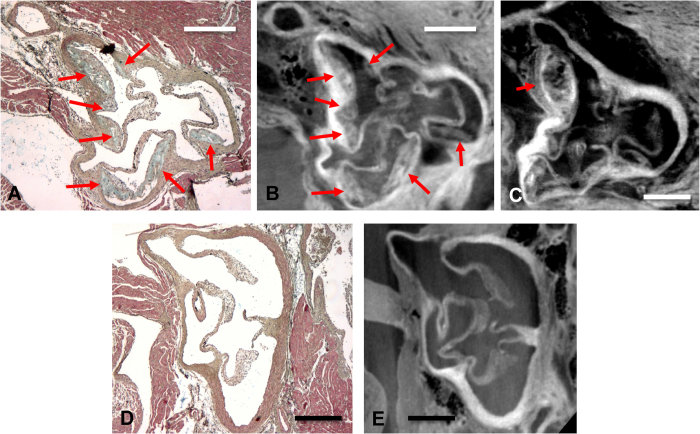
Visualization of the aortic sinus. (**A**) In a slice stained with Movat’s pentachrome, the valves and several atherosclerotic plaques of different size (arrows) can be observed. Here, blue-green stains for ground substance (non-cellular components of the extracellular matrix), blue-black stains for nuclei and elastic fibers, red stains for muscle, intense red stains for fibrinoid material and fibrin and green-yellow stains for collagen and reticulin fibers. (**B**) A DPC image at the same location that was taken from a 3D dataset allows the identification of the same structures, with sufficient contrast between the background, valves, plaques and vessel walls. (**C**) A slice from the 3D dataset with a slightly different orientation allows for an improved overview of the plaque. (**D**,**E**) Plaques cannot be observed in the Movat’s pentachrome staining or DPC image of the aortic sinus of a WT control mouse. Scale bars indicate 200 μm.

Movat’s pentachrome staining of histological cross-sections of the aortic sinus showed intermediate-stage atherosclerotic plaques, defined by the presence of necrotic cores containing cholesterol clefts and thin fibrous caps. The DPC images at the same location matched well with these histological slices (Fig. 2). The vessel wall, plaques and valves were readily observed, while the thin caps of the intermediate plaques were difficult to observe in the DPC images.

**Figure 3:**
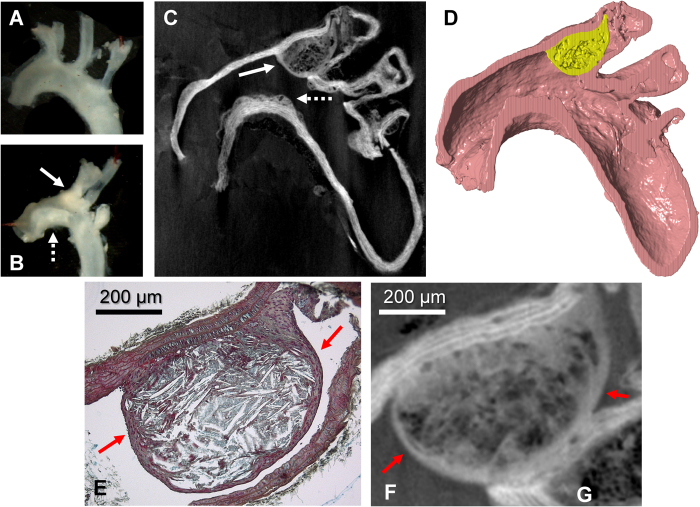
Overview of the DPC imaging and histology in the mouse aorta and branches. (**A**,**B**) *En face* photos of the excised aortas and branches of a WT control and ApoE^−/−^ mouse. In the latter, plaques can be clearly observed in the inner curvature of the aorta and the brachiocephalic artery (arrows). (**C**) DPC image of the same mouse aorta as in B). A large plaque that obstructs most of the brachiocephalic artery (solid arrow) and small plaques on the inner curvature (dotted arrow) can be clearly observed. (**D**) Longitudinal view of a 3D segmentation of the plaque within the brachiocephalic artery; the volume of the plaque (yellow) was calculated to be 209 nL. (**E**) Movat’s pentachrome staining of the same large brachiocephalic plaque. While the plaque obstructs most of the artery and contains a large necrotic core with numerous cholesterol clefts, its overlying cap (arrows) is fairly thick. (**F**) Zoom-in of the DPC image at the same location, where the cap with a thickness of 28.7 ± 8.7 μm can also be observed (arrows). Although different intensities can be observed inside the plaque, the exact makeup is harder to discern.

DPC images of the aortic arches and their branches also matched well with the *en face* photos (Fig. 3). Atherosclerotic plaques with different sizes could be recognized (Fig. 3C) and segmented (Fig. 3D). In large plaques, the fibrous cap could be discerned from the necrotic core, and individual layers of smooth muscle cells in the media could be differentiated (Fig. 3E,F). However, individual cholesterol crystals that were visible in the histological sections could not readily be appreciated in the DPC images.

### Comparison of the coronary arteries in atherosclerotic and control mice

**Figure 4:**
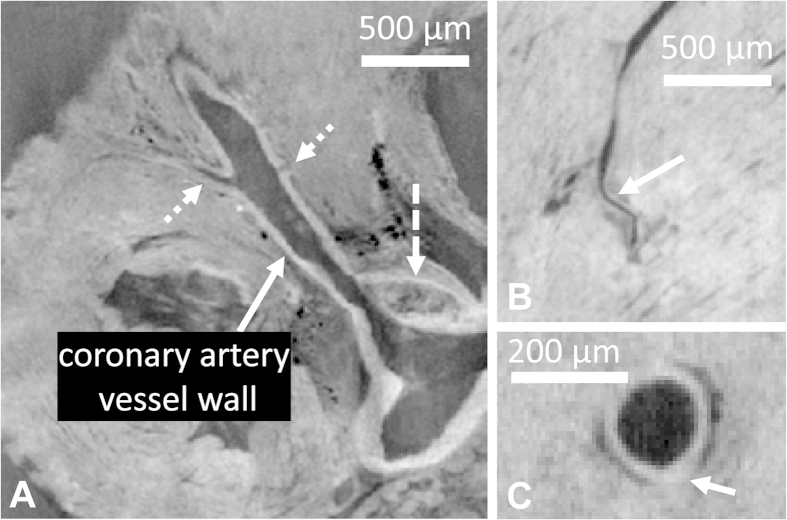
DPC images of the coronary arteries. (**A**) The left main coronary artery as it leaves the aortic root. The contrast between the vessel wall and the surrounding tissue can be clearly observed. A plaque in the aortic sinus (dashed arrow) and two branches (dotted arrows) are also visible. (**B**) In the distal part of a left coronary artery, the vessel wall can still be discerned. (**C**) In an ApoE^−/−^ mouse, a local thickening of the coronary artery wall is occasionally observed, although no plaques were found inside the coronary arteries.

**Figure 5:**
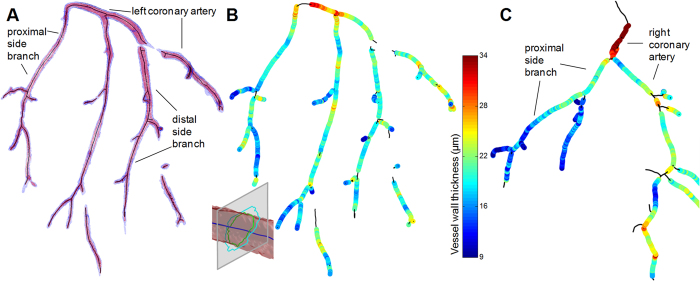
Vessel wall thickness of the mouse coronary arteries. (**A**) Segmentations of the inner (purple) and outer (blue) vessel wall of the left coronary artery system of an ApoE^−/−^ mouse with their centerlines (black). Regions where the differentiation was unclear or where the vessel had collapsed were not segmented. The inset shows an example of the subsequent determination of the vessel wall thickness, where a gray plane perpendicular to the centerline is used to determine the distance between the inner and outer vessel wall, shown as contours. (**B**) The determined vessel wall thickness in the same mouse projected on top of the centerlines, with the color bar indicating the thickness in μm. While the thickness appears to decrease as the vessels advance from the root, there is a considerable variability. (**C**) Vessel wall thickness of the right coronary system of a WT control mouse. While several focal points of thickness can be observed, in general the vessel wall thickness appears to have less variability than those of their ApoE^−/−^ counterparts.

DPC imaging provided good contrast between the coronary artery lumen, the coronary artery wall and the surrounding parenchyma (Fig. 4). The SNR of the vessel wall was 139.7 ± 2.8, the SNR of the lumen was 120.1 ± 7.3, and the SNR of the neighboring parenchyma was 134.2 ± 3.1, resulting in a vessel-wall-to-lumen CNR of 18.4 and a vessel-wall-to-parenchyma CNR of 5.5. While several plaques were observed near the ostia of the coronary arteries, no plaques were observed in more distal segments. However, focal wall thickening (Fig. 4C) was regularly observed along the coronary arteries of the atherosclerotic mice, while no such thickening was observed in the control mice. Segmentation of the coronary vessel wall was robust and resulted in detailed overviews of the distribution of the vessel wall thickness (Fig. 5). Several differences were found between the coronary segmental wall thickness of the ApoE^−/−^ mice and that of the WT control mice (Table 1), although the vessel wall thickness of neither group was consistently larger. The standard deviation of the wall thickness was mostly higher in the ApoE^−/−^ than in the WT control mice, consistent with the observations of focal wall thickening.

## Discussion

The *ex-vivo* DPC images of the hearts and aortas of ApoE^−/−^ mice gave a clear 3D overview of the large structures, while microscopic structures such as the coronary artery wall, atherosclerotic plaques, and chordae tendineae were visualized with high contrast and detail. The 3D nature of the imaging technique furthermore allowed for the reassessment of any detail from different angles, which was especially useful for tortuous or complicated structures such as the coronary arteries and the heart valves. This can for example be used to accurately assess the total plaque burden in atherosclerosis or to create anatomical atlases of the mouse heart^18^.

The 3D DPC modality had several unique features when compared to other modalities. Slices from the 3D DPC image datasets were matched to Movat’s pentachrome histological staining, which saw good agreement between the two modalities, both on the dimensions and visibility of atherosclerotic plaque structures. Since the resolution of the DPC imaging was set to ~15 μm in all directions, it was not possible to visualize structures smaller than this resolution, such as the individual cholesterol crystals that were visible on the histological slices. However, in a previous phase-contrast imaging study of significantly smaller samples it was demonstrated that the mass density of the different components of atherosclerotic plaque could be robustly differentiated and matched to histology^19^. A similar or higher level of detail in the identification of different atherosclerotic plaque components may be obtained with two-photon laser scanning microscopy (TPLSM)^20^ or nonlinear optical (NLO) microscopy^21^,^22^. These techniques offer a higher spatial resolution (down to ~0.3 μm) for 3D analysis of sub-cellular components when compared to DPC imaging. However, the field-of-view is significantly smaller for optical microscopy and the visualization of the entire coronary tree of a mouse heart may currently not be feasible.

The topology of the coronary arteries of both the ApoE^−/−^ and WT control mice agreed very well with previously described coronary systems^23^. Small but significant differences in average vessel wall thickness were found between the two groups, although increasing the group size beyond the demonstrative number used in this study might allow for a better assessment of such differences. However, atherosclerosis progression is a regional and heterogeneous process and a systemic increase of the coronary artery vessel wall thickness may not necessarily be expected in ApoE^−/−^ mice of this age^1^: coronary plaques have only been observed in ApoE^−/−^ mice that were more than twice as old as those used in this study^24^.

Unlike in humans, the mouse coronary arteries do not lie on the surface of the heart, but are embedded within the myocardium. The contrast between the vessel wall and the parenchyma therefore made automatic segmentation challenging, most likely due to both tissues consisting of different types of muscle cells. However, this contrast still allowed for straightforward semi-automatic segmentation. Possible applications of such a 3D overview of the coronary vessel wall include for example studies of coronary endothelial remodeling in mouse models or the study of differences in the vascular topology.

There were several limitations to this study. Firstly, access to a synchrotron beamline with a DPC setup may not be straightforward, although phase-contrast infrastructure is available at several facilities. The translation of the technique to the clinical setting, albeit very limited, looks promising though: *ex-vivo* synchrotron DPC mammography has already been demonstrated to be superior to X-ray mammography^17^, while grating-based phase-contrast CT with a regular X-ray source was recently performed on excised human carotids with a resolution of 100 μm^25^. Secondly, a low number of animals were scanned: it was sufficient to illustrate the possibilities of the technique for atherosclerosis detection, but for in-depth studies of for example coronary vascular remodeling, a higher number of animals will be needed. Thirdly, while the acquired spatial resolution (15 μm) resulted in an accurate depiction of the overall anatomical structures, the vessel wall thickness measurement for mid-ventricular and distal side branches is close to this spatial resolution. To characterize the accuracy of the vessel wall thickness measurement, a higher number of samples followed by a comparison with histology may be needed.

We conclude that differential phase contrast imaging of the cardiovascular system of atherosclerotic mice allows for a detailed 3D morphological assessment of both large structures and microscopic details of atherosclerotic burden.

## Materials and Methods

### Animal Protocol and Sample Preparation

All animal experiments were approved by the animal ethics committee of the canton of Vaud, Switzerland, and all animal experiments were carried out in accordance with the approved guidelines. Two ApoE^−/−^ mice and two weight-matched wild-type (WT) control mice (10 and 5 weeks old, respectively, both on a C57BL/6 background, from Charles River Laboratories, L’Arbresle, France) were fed a high-fat diet (20% milk butter, 0.15% cholesterol; Western Diet U8958, Safe Diets, Augy, France) for 12 weeks. They were then euthanized through pentobarbital overdose (at ages 22 and 17 weeks, weights 29.9 ± 0.7 g and 29.9 ± 0.4 g, respectively). Their hearts, aortic arches with branching vessels and thoracic aortas were excised and perfused with saline, which should among others increase the contrast between the lumen and vessel wall, after which they were fixed in a 5% formalin solution. After 1 week in the formalin solution, the hearts and aortas were separately fixed in a 2% agarose gel within 1.5 ml tubes.

### Differential Phase Contrast Imaging

The DPC setup was implemented as previously described^14^. Briefly, the TOMCAT (TOmographic Microscopy and Coherent rAdiology experimenTs) beamline at the Swiss Light Source (Villigen, Switzerland) receives 25 keV synchrotron X-rays from a 2.9 T bending magnet. The beam passes through a monochromator before passing through the sample, which is mounted on a rotating stage. Finally, it passes through two gratings that are placed at a fractional Talbot distance^26^ of 120 mm of each other. During acquisition, the sample is rotated over 180° in 1000 steps. At 25 m from the source, the X-ray beam measures 15.2 mm horizontally by 3.7 mm vertically (thus defining the maximum field of view). This setup of the optics resulted in an isotropic spatial resolution of ~15 μm. The scan time of this imaging volume was ~2 hours.

Each sample was scanned once. Raw 3D datasets were acquired and reconstructed with an isotropic voxel size of [7.4 μm]^3^ (i.e. at approximately half the spatial resolution). The datasets were then transferred to a PC where any striping and ringing artifacts were removed with a combined wavelet-Fourier filter^27^ in Matlab (Mathworks, Natick, MA, USA).

### DPC Image Visualization, Segmentation and Processing

3D image volumes were assessed in Fiji^28^, where they could be reformatted as images in any oblique orientation of interest. The inner and outer surfaces of the vessel wall of the aortas and coronary arteries were semi-automatically segmented in Matlab through manual correction of a thresholded mask. Large aortic plaques were then separately segmented, and their volume was calculated.

The vessel wall thickness of the segmented coronary arteries was determined in an automated manner: first, the center line of the coronary lumen was defined through a flow model^29^. Next, on a plane perpendicular to this center line, contours of the intersecting inner and outer vessel wall surfaces were drawn. For each center line point, the average radii *r* of the inner and outer contours were approximated as *r = √(Contour Area/π)*. These radii were subtracted to obtain an estimate of the vessel wall thickness at each center line point. This vessel wall thickness was measured in equidistant steps of 22.2 μm along the center lines and was grouped into the proximal, mid and distal segments of the main coronary arteries and of the distal branches, resulting in at least 100 vessel wall thickness measurements per segment type.

The signal-to-noise ratio (SNR) and contrast-to-noise ratio (CNR) of the coronary vessel wall were defined as S_wall_/N and (S_wall_-S_parench_)/N, respectively. S_wall_, S_lumen_ and S_parench_ were the average signal in a manually drawn region of interest in the vessel wall, lumen and adjacent parenchyma, respectively, while the noise N was defined as the standard deviation of a homogeneous empty region outside the heart.

### Histology

The samples were subsequently embedded in paraffin and sectioned in 3 μm slices at the level of atherosclerotic plaques in the aortic sinus and brachiocephalic artery. Movat’s pentachrome histological staining^30^ was then performed for plaque characterization. The histological slices were visually matched with the DPC datasets.

### Statistical Analyses

The coronary artery wall thicknesses of the ApoE^−/−^ and control mice were compared with unpaired two-tailed Student’s t-tests. *p* < 0.05 was considered significant.

## Additional Information

**How to cite this article**: Bonanno, G. *et al.* Ultra-high-resolution 3D imaging of atherosclerosis in mice with synchrotron differential phase contrast: a proof of concept study. *Sci. Rep.* **5**, 11980; doi: 10.1038/srep11980 (2015).

## Figures and Tables

**Table 1:** Vessel wall thickness of mouse coronary arteries per segment.

Vessel Segment	ApoE^−/−^ Wall thickness (μm)	WT Wall thickness (μm)
Proximal main arteries	36.7 ± 6.2	37.4 ± 5.6
Mid main arteries*	23.5 ± 3.7	20.8 ± 2.8
Distal main arteries*	21.3 ± 2.8	20.6 ± 4.7
Proximal side branches*	18.9 ± 5.1	21.1 ± 2.4
Mid side branches*	20.2 ± 2.9	20.9 ± 2.1
Distal side branches*	17.0 ± 3.6	13.6 ± 3.3

Here *indicates p < 0.05 for the Student’s *t*-test between the ApoE^−/−^ and WT mouse coronary arteries.
